# Is the neighborhood of interaction in human crowds metric, topological, or visual?

**DOI:** 10.1093/pnasnexus/pgad118

**Published:** 2023-05-16

**Authors:** Trenton D Wirth, Gregory C Dachner, Kevin W Rio, William H Warren

**Affiliations:** Department of Cognitive Linguistic and Psychological Sciences, Brown University, Providence, RI 02912, USA; Department of Biology, Northeastern University, Boston, MA 02115, USA; Department of Cognitive Linguistic and Psychological Sciences, Brown University, Providence, RI 02912, USA; Reality Labs, Meta, Redmond, WA 98052, USA; Department of Cognitive Linguistic and Psychological Sciences, Brown University, Providence, RI 02912, USA; Department of Cognitive Linguistic and Psychological Sciences, Brown University, Providence, RI 02912, USA

**Keywords:** collective behavior, crowd dynamics, pedestrian dynamics, agent-based model, self-organization

## Abstract

Global patterns of collective motion in bird flocks, fish schools, and human crowds are thought to emerge from local interactions within a neighborhood of interaction, the zone in which an individual is influenced by their neighbors. Both metric and topological neighborhoods have been reported in animal groups, but this question has not been addressed for human crowds. The answer has important implications for modeling crowd behavior and predicting crowd disasters such as jams, crushes, and stampedes. In a *metric neighborhood*, an individual is influenced by all neighbors within a fixed radius, whereas in a *topological neighborhood*, an individual is influenced by a fixed number of nearest neighbors, regardless of their physical distance. A recently proposed alternative is a *visual neighborhood*, in which an individual is influenced by the optical motions of all visible neighbors. We test these hypotheses experimentally by asking participants to walk in real and virtual crowds and manipulating the crowd's density. Our results rule out a topological neighborhood, are approximated by a metric neighborhood, but are best explained by a visual neighborhood that has elements of both. We conclude that the neighborhood of interaction in human crowds follows naturally from the laws of optics and suggest that previously observed “topological” and “metric” interactions might be a consequence of the visual neighborhood.

**Significance Statement**

Patterns of “flocking” or collective motion in animals and humans emerge from local interactions between individuals in a neighborhood of interaction. Some species appear to interact with all neighbors within a fixed distance, in a *metric neighborhood*, whereas others seem to interact with a fixed number of nearest neighbors in a *topological neighborhood*. Alternatively, interactions in a *visual neighborhood* would be guided by the optical motions of visible neighbors. We experimentally tested these hypotheses for humans by asking participants to walk in real and virtual crowds. The results rule out a topological neighborhood, are closer to a metric neighborhood, but are best explained by a visual neighborhood. This finding has important applications to modeling crowd dynamics and predicting crowd disasters.

## Introduction

Large-scale patterns of coordinated motion are observed in many animal groups, including flocks of birds, schools of fish, herds of mammals, and crowds of humans (1–5,). It is widely believed that such global patterns of collective motion emerge from many local interactions between individuals in a process of self-organization (1, 6, 7). Understanding collective motion thus depends on characterizing these local interactions (8, 9). First, what are the *rules of engagement* that govern how an individual interacts with a neighbor? Second, what is the *neighborhood of interaction* over which these rules operate and the influences of multiple neighbors are combined? Here we aim to characterize the neighborhood of interaction in human crowds.

**Fig. 1.**
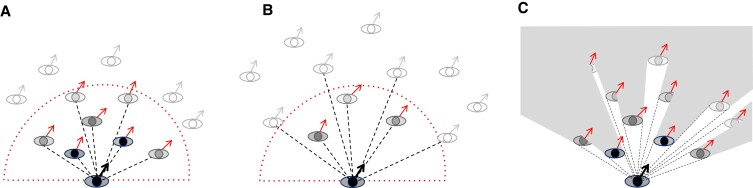
Testing the metric, topological, and visual hypotheses. A) High density: Soft metric neighborhood (dotted boundary) predicts decreasing influence of neighbors (shading) with metric distance from a pedestrian (bottom), whereas topological neighborhood (dashed lines) predicts decreasing influence with a neighbor's ordinal rank. Metric and topological distances are correlated here. B) Low density: The hypotheses are dissociated by manipulating crowd density. The metric neighborhood predicts that increasing distance will weaken neighbor influence, whereas the topological neighborhood predicts their influence will remain constant. C) In a visual neighborhood, influence decreases with both metric distance and visual occlusion. Contrary to the topological model, influence depends on density; contrary to the metric hypothesis, the model generalizes to crowds with different densities and configurations. Modified from Dachner, et al., 2022 with permission under the guidelines of Royal Society Publishing.

Many mathematical models of collective motion assume rules of engagement based on hypothesized forces of attraction, repulsion, and velocity alignment (10–14). Such models—including our own (15)—typically average the influence of all neighbors within a *metric neighborhood* or zone of fixed radius (Fig. 1A, dotted boundary), with neighbor influence often decreasing with metric distance (15–17). In contrast, others have proposed a *topological neighborhood* (18–22) (Fig. 1A, dashed lines) in which an individual is influenced by a fixed number of nearest neighbors, regardless of their metric distance, and neighbor influence may decrease with ordinal rank. All of these models can be described as “omniscient” because they assume the physical positions and velocities of all neighbors as input. We compare them with a new *visual neighborhood* model (23) (Fig. 1C) based on an embedded view in a crowd (24–28), with elements of both metric and topological distances (29).

Evaluating these hypotheses is nontrivial, for metric distance (number of meters) and topological distance (ordinal rank) are naturally correlated. Yet the two hypotheses can be dissociated by varying group density (Fig. 1A and B). The metric hypothesis predicts that velocity alignment should depend on density, because the influence of neighbors decreases with their physical distance (shading). In contrast, the topological hypothesis predicts no effect of changes in density, because neighbor influence only depends on ordinal rank (dashed lines). Finally, the visual hypothesis (Fig. 1C) predicts that alignment should depend on both the distance and configuration of neighbors, which determine optical velocities and visual occlusion (23).

Observational studies of bird flocks have found empirical support for the first two hypotheses. Starlings appear to possess a topological neighborhood (2, 18), for the ordinal range of interaction remains constant at six to seven neighbors despite natural fluctuations in flock density. In contrast, roosting chimney swifts appear to have a metric neighborhood (30), for alignment is maximal at 1.4 m despite variations in density, and alignment with the *n*th nearest neighbor depends on its metric distance (see also (31)). Alternatively, in fish schools, a study of golden shiners found that data were best fit by a visual neighborhood (25). Other analyses of small groups of mosquito fish and rummy-nose tetra have found that individuals respond to just one or two neighbors at a time, who may change with shifts of attention (32–34); modeling shows that this strategy is sufficient to generate collective motion (24, 34, 35). Humans, however, have been found to combine the influences of many neighbors (6, 36), independent of attention (37), and thus we do not pursue the latter hypothesis.

To date, the metric and topological hypotheses have not been tested in humans, for the existing data do not distinguish them. The answer is of central importance for modeling crowd dynamics, simulating emergency evacuations, and predicting crowd disasters such as jams, crushes, and stampedes (3, 38–41). We test the hypotheses experimentally by manipulating the density of virtual and real crowds, perturbing the heading (walking direction) of a subset of neighbors, and measuring the participant's heading response. The metric and visual hypotheses predict that varying the density of neighbors will influence the heading response, whereas the topological hypothesis predicts that density will have no effect.

Specifically, we manipulated the distances of perturbed and unperturbed neighbors in a virtual crowd so that the metric hypothesis predicts a stronger (first experiment) or weaker (second experiment) heading response with a higher density. We find significant effects of density in the predicted directions. To generalize these results to real crowds (third experiment), we manipulated the density of human “swarms” and analyzed the degree of alignment. We find greater alignment in high-density swarms, whether plotted as a function of metric or topological distance.

These findings rule out a strictly topological neighborhood. The direction of the density effect is predicted by a metric neighborhood model (15), but the quantitative results are best predicted by a visual model (23). We conclude that the neighborhood of interaction in humans is not topological, depends on metric distance, and is best explained by visual information.

## Results

We begin by describing models of metric, topological, and visual neighborhoods, then test them experimentally.

### Neighborhood models

#### Metric model

To describe a metric neighborhood, we used our empirical model of local interactions in human crowds (15). The rules of engagement were derived from experiments on following in pairs of pedestrians, which found that the follower matches the heading direction and speed of the leader. The neighborhood of interaction was derived from previous experiments showing that a pedestrian is influenced by a weighted average of neighbors (Eqs. 1a and 1b), where the weight decays exponentially with metric distance (Eq. 1c):


(1a) $${\ddot{\phi }}_{p}=-\frac{k}{n}\overset{n}{\underset{i=1}{\sum }}\;w_{i}\sin(\phi_{p}-\phi_{i})$$

(1b) $${\ddot{r}}_{p}=-\frac{c}{n}\overset{n}{\underset{i=1}{\sum }}\;w_{i}({\dot{r}}_{p}-{\dot{r}}_{i})$$

(1c) $$w_{i}=\frac{a}{e^{\omega d_{i}}+a}$$

To control heading (Eq. 1a), a pedestrian *p*'s angular acceleration (${\ddot{\phi }}_{p}$) is proportional to the mean difference between *p*'s current heading ($\phi_{p}$) and that of each neighbor ($\phi_{i}$), where *n* is the number of neighbors within a 5-m radius and a 180° field of view. To control speed (Eq. 1b), an analogous equation governs *p*'s radial acceleration (${\ddot{r}}_{p}$). The coupling strength parameters *k* = 3.15 and *c* = 3.61 were fit to previous data on pedestrian following (42). The weight of each neighbor *w_i_* (Eq. 1c) decreases as a logistic function of metric distance *d_i_*, where the decay rate *ω*=1.3 and constant *a* = 9.2 were fit to a sample of human “swarm” data (15). These parameter values were held fixed in the present simulations.

This results in a metric neighborhood with a “soft” radius that asymptotes to zero around 4–5 m, defining the range of interaction (dotted boundary in Fig. 1). According to the model, *p*'s heading direction stabilizes on the mean heading and speed in the neighborhood. The physical proximity of neighbors determines the strength of attraction and hence the turning rate and relaxation time of the heading response.

#### Topological model

A topological neighborhood is similarly based on a weighted average of neighbors (Eqs. 1a and 1b), but the weight decays linearly with the topological distance of each neighbor (ordinal rank *R_i_*) rather than metric distance:


(2) $$w_{R_{i}}=mR_{i}+b$$

We used linear regression to fit the data in the high-density “swarm” condition, yielding slope *m* = −0.07 and intercept *b* = 1.03 (*R*^2^ = 0.97) (see Supplementary Data S3 and Fig. S5). The topological hypothesis holds that this decay rate is independent of density.

#### Visual model

The visual model (23) is also based on a weighted average of neighbors, but it replaces omniscient variables (neighbor distance or rank, heading, and speed) with visual variables (angular velocity, optical expansion, and visibility):


(3a) $${\ddot{\phi }}_{p}=\frac{1}{n}\overset{n}{\underset{i=1}{\sum }}\;v_{i}[c_{1}(\cos\;\beta_{i}){\dot{\psi }}_{i}-c_{2}(\sin\;\beta_{i}){\dot{\theta }}_{i}]$$

(3b) $${\ddot{r}}_{p}=\frac{1}{n}\overset{n}{\underset{i=1}{\sum }}\;v_{i}[-\;c_{3}(\sin\;\beta_{i}){\dot{\psi }}_{i}-c_{4}(\cos\;\beta_{i}){\dot{\theta }}_{i}]$$

Specifically, pedestrian *p*'s heading is controlled by canceling the angular velocity ${\dot{\psi }}_{i}$ and expansion rate ${\dot{\theta }}_{i}$ (rate of change in visual angle) of all visible neighbors (i=1…n). These two optical variables trade off as cosine and sine functions of the neighbor's eccentricity $\beta_{i}$ in the field of view, which is centered on *p*'s heading direction. For example, if a neighbor directly ahead of *p* turns right, this generates a positive angular velocity but little optical expansion, whereas if a neighbor on *p*'s left turns right, this generates a positive optical expansion but little angular velocity (see ref. (23) for details). A complementary equation controls *p*'s speed (Eq. 3b). The constants $c_{1}=14.38$, $\;c_{2}=59.71$, $c_{3}=0.18$, and $c_{4}=0.72$ were fit to previous data on pedestrian following (43) and held fixed.

Critically, optical velocities (${\dot{\psi }}_{i},{\dot{\theta }}_{i}$) decrease with metric distance *d* as $\mathrm{ta}n^{-1}(1/d_{i})$, in accordance with Euclid's law of visual angle, thus eliminating an explicit distance term (Eq. 1c). In addition, nearer neighbors tend to visually occlude farther neighbors, depending on their visual directions, ordinal ranks, and metric separation in depth (25, 29, 44). The model weights each neighbor in proportion to their *visibility*, which ranges from $v_{i}=0$ (fully occluded) to $v_{i}=1$ (fully visible); neighbors below a visibility threshold $v_{t}<0.15$ (fit to experimental data) are set to zero, and *n* is the number of neighbors at or above threshold. The neighborhood's range of interaction is not a fixed radius, but limited by the complete occlusion of farther neighbors, which varies with density and crowd configuration (29).

The visual model thus depends on both metric and topological distances, but the neighborhood of interaction is determined by the laws of optics. The model stabilizes on the mean heading and speed in the visual neighborhood, and the attraction strength, turning rate, and relaxation time are determined by the visibility of neighbors and the magnitudes of their optical motions.

### Experiments in virtual crowds

We tested the neighborhood hypotheses by varying the density of a virtual crowd. This allowed us to manipulate the behavior of virtual neighbors, who moved on prescribed paths, and measure their influence on a participant's walking trajectory. Participants walked freely in a 12 m × 14 m area while viewing a group of 12 virtual humans in a mobile virtual reality headset. We asked participants to walk with the virtual crowd and treat them as if they were real people. During each trial, we perturbed the heading (walking direction) of a subset of virtual neighbors, all to the left (−10°) or to the right (+10°), and recorded the participant's walking direction (the “heading response”).

**Fig. 2.**
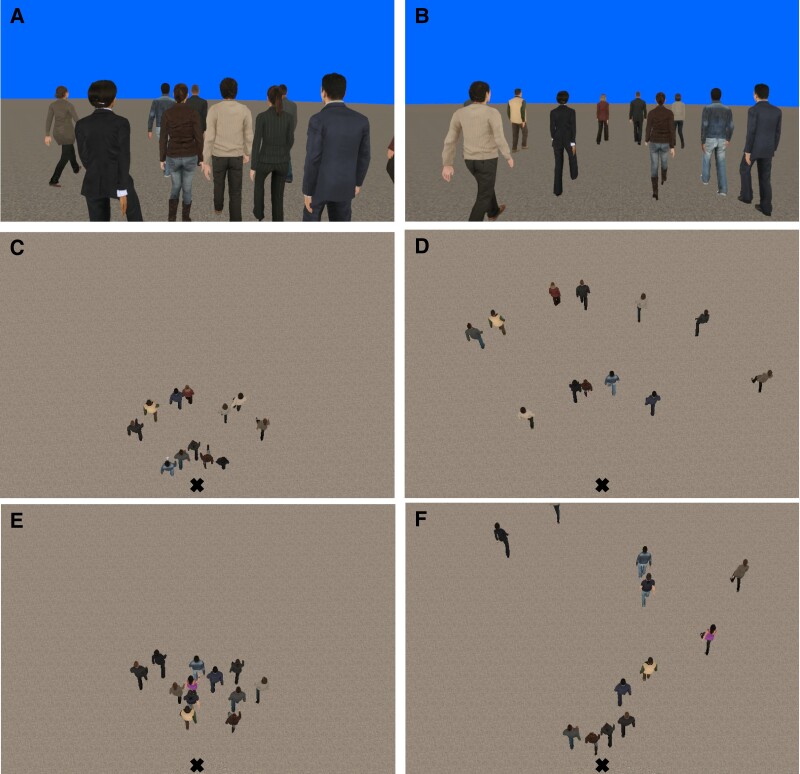
Virtual crowd displays. Participant's view in the A) high-density and B) low-density conditions of the first experiment. Bird's-eye view in the C) high-density and D) low-density conditions of the first experiment and E, F) the second experiment. “X” indicates the participant's position.

High- and low-density crowds were created by positioning virtual humans at prescribed initial distances from the participant and then randomly jittering their positions (Fig. 2). On each trial, the virtual crowd appeared with their backs to the participant (Fig. 2A and B); after 1 s, a verbal “begin” command was played and the crowd accelerated forward for 3 s to a walking speed of 1.0 m/s; 2 s later, the subset's heading was perturbed, and the display continued for another 8 s. The participant's head position in the horizontal plane was recorded, filtered, and used to compute the time series of heading for each trial. The final heading on each trial was the mean value between 4 and 6 s postperturbation. A mean time series was computed for each participant in each condition for analysis.

#### Heading responses increase with density when random neighbors are perturbed

In the first experiment, the heading of a random subset of the virtual neighbors (0, 3, 6, 9, or all 12) was perturbed (±10°) on each trial. In the high-density condition, five neighbors were initially positioned at 1.5 m and seven at 3.5 m (Fig. 2C); in the low-density condition, the initial distances were 3.5 and 7.5 m (Fig. 2D). Each participant (*N* = 10) received 8 trials in each of the 10 conditions. Speed was perturbed (±0.3 m/s) in a separate condition and yielded similar results (see Supplementary Data S1 and Fig. S1).

**Fig. 3.**
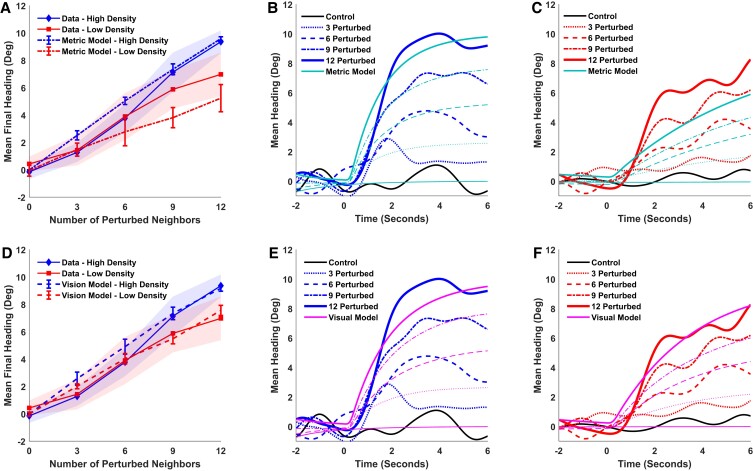
Results and simulations of the first experiment. Top row, metric model: A) mean final heading for humans and metric model as a function of the number of perturbed neighbors. Shaded regions represent the 95% CI of the human data. The error bars in plots A) and D) represent the 95% CI for the model. B) High-density condition: mean time series of heading for human data and metric model; curves represent the number of perturbed neighbors. C) Low-density condition: same. Bottom row, visual model: D–F) same data with visual simulations.

According to the metric model (Eq. 1), the participant is attracted to the mean heading in the neighborhood, which increases with the percentage of perturbed neighbors. Because nearer neighbors have higher weights, the model predicts that the attraction strength will be greater, the turning rate faster, and the relaxation time shorter at higher density. Consequently, the mean final heading after 4–6 s should be larger in the high-density condition than the low-density condition, and this difference should increase with the percentage of perturbed neighbors (Fig. 3A, dotted curves). In contrast, the topological model predicts no difference between the high- and low-density conditions (see Supplementary Data S1 and Fig. S2).

The results appear in Fig. 3A (solid curves). As the number of perturbed neighbors increases, mean final heading becomes larger in the high-density condition (blue) than the low-density condition (red). A linear mixed effects (LME) regression analysis found that this interaction was significant: the effect of density increased with the number of perturbed neighbors (*χ*^2^(1) = 6.111, *P* = 0.0134). This significant dependence on density is contrary to the topological hypothesis. The metric model (dotted curves) is closer to the human data, although it only lies within the 95% CI in four of the eight perturbation conditions, and undershoots the data in the low-density condition (red).

Attraction strength is indicated by the time series of heading: the postperturbation slopes are steeper in the high-density condition (Fig. 3B, blue curves) than the low-density condition (Fig. 3C, red curves), increasingly so as more neighbors are perturbed. An LME regression showed that this three-way interaction (density × time × perturbed neighbors) is significant (*χ*^2^(1) = 4.163, *P* = 0.041), confirming that the turning rate is faster in the high-density condition, as predicted by the metric model. In contrast, the significant dependence on density is inconsistent with the topological hypothesis. (Regression results appear in Tables S1 and S4.)

##### Model simulations

To compare the metric model (Eq. 1) with the human data quantitatively, we simulated each experimental trial with no free parameters. The model agent was initialized with the participant's position and heading 2 s before the perturbation, the distance and heading of virtual neighbors were taken as input on each time step, and a time series of the agent's heading was computed. We then calculated the agent's mean time series in each condition and compared it with the participant's mean time series in the corresponding condition using the root of the mean squared error (RMSE).

Time series of heading for the metric model (cyan curves) are plotted together with the human data in Fig. 3B (high-density condition) and Fig. 3C (low-density condition). The model again appears to undershoot the data at low density. The mean RMSE_m_ for perturbation trials was 2.06°.

We repeated these simulations using the visual model (Eq. 3). In this case, the input to the model agent was the angular velocity, expansion rate, eccentricity, and visibility of each neighbor in the participant's field of view, calculated from their position, heading, and speed at each time step. The model's mean final heading (Fig. 3D, dashed curves) is closer to the human data, particularly in the low-density condition (red curves), and is within the 95% CI of the data in six of the eight perturbation conditions. The mean time series for the visual model are plotted together with the human data in Fig. 3E (high density) and Fig. 3F (low density). The mean RMSE_v_ is 1.96°, closer to the human data than the metric model. To compare the relative strength of evidence for the two models, we computed Bayes factors, yielding anecdotal evidence favoring the visual model overall (BF_vm_ = 1.42), with substantial evidence in the low-density condition (BF_vm_ = 8.85). The visual model thus explains the human data as well as or better than the metric model.

Is this good model performance? Given the inherent noise in the data due to gait oscillations and measurement error, we estimated the limit on best performance by computing the RMSE between the participant mean time series in the control condition (0 perturbed neighbors) and a heading of 0°. This yielded a mean RMSE of 1.21°, indicating that the visual model is only 0.75° from the limit, although not perfect (BF_1v_ > 100). Conversely, to estimate the worst performance for a model that does not respond to the input, we computed the RMSE between the participant mean time series in the perturbation conditions and a heading of 0°. This yielded a mean RMSE of 3.98°, indicating that visual model is much better than doing nothing (BF_v0_ >> 100). The visual model is thus near the high end of possible model performance, close to the human data.

##### Conclusion

The first experiment finds that participants have a stronger heading response in a higher-density crowd. Specifically, when perturbed neighbors are in the majority and in closer proximity to the participant, they exert a greater influence, producing a faster turning rate and a larger final heading. This significant density dependence contradicts the topological hypothesis, which predicts that density should have no effect. The direction of the density effect is consistent with the metric hypothesis, but the data are better predicted by the visual model.

##### Heading responses decrease with density when nearest neighbors are perturbed

The first experiment found that the heading response increased with crowd density. But if the response depends on metric distance, we should be able to manipulate the proximity of unperturbed neighbors to elicit the opposite effect: a *decrease* in the heading response with higher density. The second experiment tested this prediction. Specifically, we held the metric distances of the four nearest neighbors’ constant and varied density by manipulating the distances of the other eight neighbors (Fig. 2E and F). When the near neighbors are perturbed, the metric hypothesis predicts a weaker response in the high-density condition than the low-density condition. In contrast, the topological hypothesis predicts that the distance of the unperturbed neighbors should have no effect.

In this experiment, the heading of the nearest neighbors (0, 2, or 4) was always perturbed (±10°). The four nearest neighbors were positioned at fixed distances (1.5, 1.7, 1.9, and 2.1 m) before jittering, while the remaining eight neighbors appeared at moderate distances in the high-density condition (2.3 to 3.7 m, Fig. 2E), and far distances in the low-density condition (3.1 to 11.1 m, Fig. 2F), and were never perturbed. Each participant (*N* = 12) received 16 trials in each of the 6 conditions. The speed of the virtual crowd was increased slightly to a more comfortable walking speed (1.15 m/s), so the display continued for 5.4 s postperturbation and mean final heading was recorded between 2.4 and 4.4 s. Otherwise, the procedure was the same as before.

The metric model predicts that the heading response should be reduced in the high-density condition, because the unperturbed neighbors are closer and more influential. In contrast, in the low-density condition, the unperturbed neighbors are farther away and less influential, so the response to the perturbed neighbors should be stronger, yielding a faster turning rate and a larger final heading. In other words, the density effects should be the opposite of those observed in the first experiment.

**Fig. 4.**
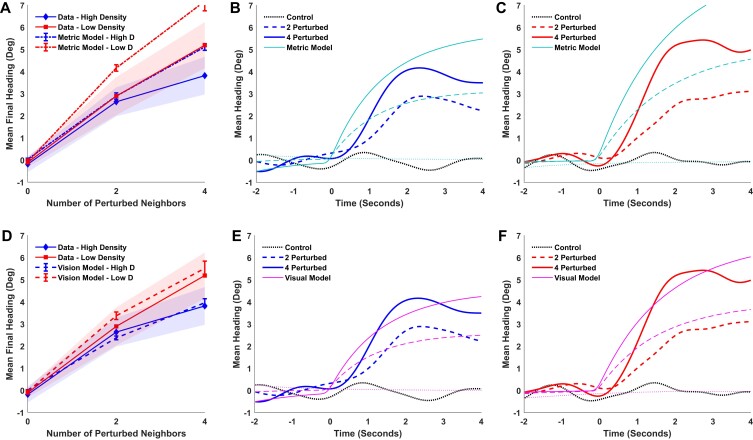
Results and simulations of the second experiment. Top row, metric model: A) mean final heading for humans and metric model as a function of the number of perturbed neighbors. Shaded regions represent the 95% CI of the human data. The error bars in plots A) and D) represent the 95% CI for the model. B) High-density condition: mean time series of heading for human data and metric model; curves represent the number of perturbed neighbors. C) Low-density condition: same. Bottom row, visual model: D–F) same data with visual simulations.

The results for mean final heading appear in Fig. 4A. It is clear that the density effect is reversed: final heading is now smaller in the high-density (solid blue curve) than the low-density condition (solid red curve). An LME regression confirmed a significant two-way interaction, such that the effect of density grows with the number of perturbed neighbors (*χ*^2^(1) = 5.54, *P* = 0.0186). This finding is similar to the first experiment but in the opposite direction, as expected on the metric hypothesis. On the other hand, the significant density effect contradicts the topological hypothesis (See Figure S2). Yet the metric model (dotted curves) overshoots the data by a wide margin at both densities, lying outside the 95% CI in three of the four perturbation conditions.

The effect of density on attraction strength is also reversed, for the slopes of the heading time series are shallower in the high-density (Fig. 4B, blue curves) than the low-density condition (Fig. 4C, red curves). An LME regression found that the three-way interaction (density × time × number of perturbed neighbors) was significant (*χ*^2^(1) = 4.269, *P* = 0.0388), confirming a slower turning rate in the high-density condition. In sum, the density effects were reversed, contrary to the topological hypothesis, but in the direction predicted by the metric hypothesis. (Regression results appear in Tables S2 and S4.)

##### Model simulations

We simulated heading on each trial with the metric model, as before. The mean time series of heading for the model (cyan curves) are plotted together with the human data in the high-density (Fig. 4B) and low-density (Fig. 4C) conditions. The mean RMSE was 1.48° for perturbation trials (note the smaller error due to smaller turns in this experiment). Although the metric model generates the reverse density effect, it systematically overshoots the data.

Why might this be so? The metric model approximates the effect of distance with a fixed exponential decay term that was fit to a sample of human swarm data (15). However, it does not take account of the actual optical velocities and visual occlusion in a particular crowd and thus fails to generalize to other densities and configurations of neighbors. Because the visual model is predicated on these optical variables, it should generalize to the novel crowds in the second experiment.

We simulated the data with the visual model (Eq. 3), as before. The model's mean final heading appears in Fig. 4D (dashed curves). Importantly, it closely predicts the reverse density effect, falling within the 95% CI for the data in all conditions. The mean time series of heading for the model are also closer to the human data in both high-density (Fig. 4E) and low-density (Fig. 4F) conditions. Overall, the mean RMSE is 1.18° for the visual model, which is very strongly favored over the metric model (BF_vb_ = 56.1). In addition, the performance of the visual model is only 0.43° from the inherent noise limit (mean RMSE = 0.75°), if not perfect (BF_1v_ > 100), and it is much better than doing nothing (mean RMSE = 2.55°, BF_v0_ > 100). A visual neighborhood thus explains the human data better than a metric or topological neighborhood.

##### Conclusion

The second experiment found a significant density effect once again, but in the opposite direction of the first experiment. This density dependence contradicts the topological hypothesis. The reversed density effect is consistent with the metric hypothesis, but the model overshoots the data in both high- and low-density conditions. Both experiments are best explained by the visual model, which generalizes to new density and occlusion conditions because the neighborhood is based on optical variables rather than physical distance.

### Human “swarm” experiment

To test whether our findings with virtual crowds extend to real crowds, a third experiment measured alignment in human “swarms.” Three groups of participants (*N* = 10, 16, and 20) were instructed to walk about a large tracking area (14 m × 20 m), veering randomly left and right but staying together as a group, for 2-min trials. We manipulated the initial density of the group (high and low) and analyzed the difference in heading between pairs of participants.

Each group participated in two trials at each density, for a total of 12 trials. Head positions in the horizontal plane were recorded with 16 motion-capture cameras, filtered, and used to compute the heading direction of each participant in each time step. This yielded approximately 11 min of usable data (frames in which all head positions were successfully recovered). We then measured the absolute heading difference ($\left|\Delta \phi_{i,j}\right|$) and metric distance ($d_{i,j}$) between pairs of participants *i* and *j* in each time step.

#### Alignment is greater in high-density crowds

According to both the metric and topological hypotheses, the absolute heading difference between neighbors should increase with metric distance, because metric and topological distances are correlated. But the metric hypothesis predicts a smaller heading difference (greater alignment) in the high-density condition, whether the data are plotted as a function of metric or topological distance. In contrast, the topological hypothesis predicts greater alignment in the *low*-density condition when plotted as a function of metric distance, because the *n* nearest neighbors interact over longer distances. But any effect of density should disappear when the data are plotted as a function of topological distance.

**Fig. 5.**
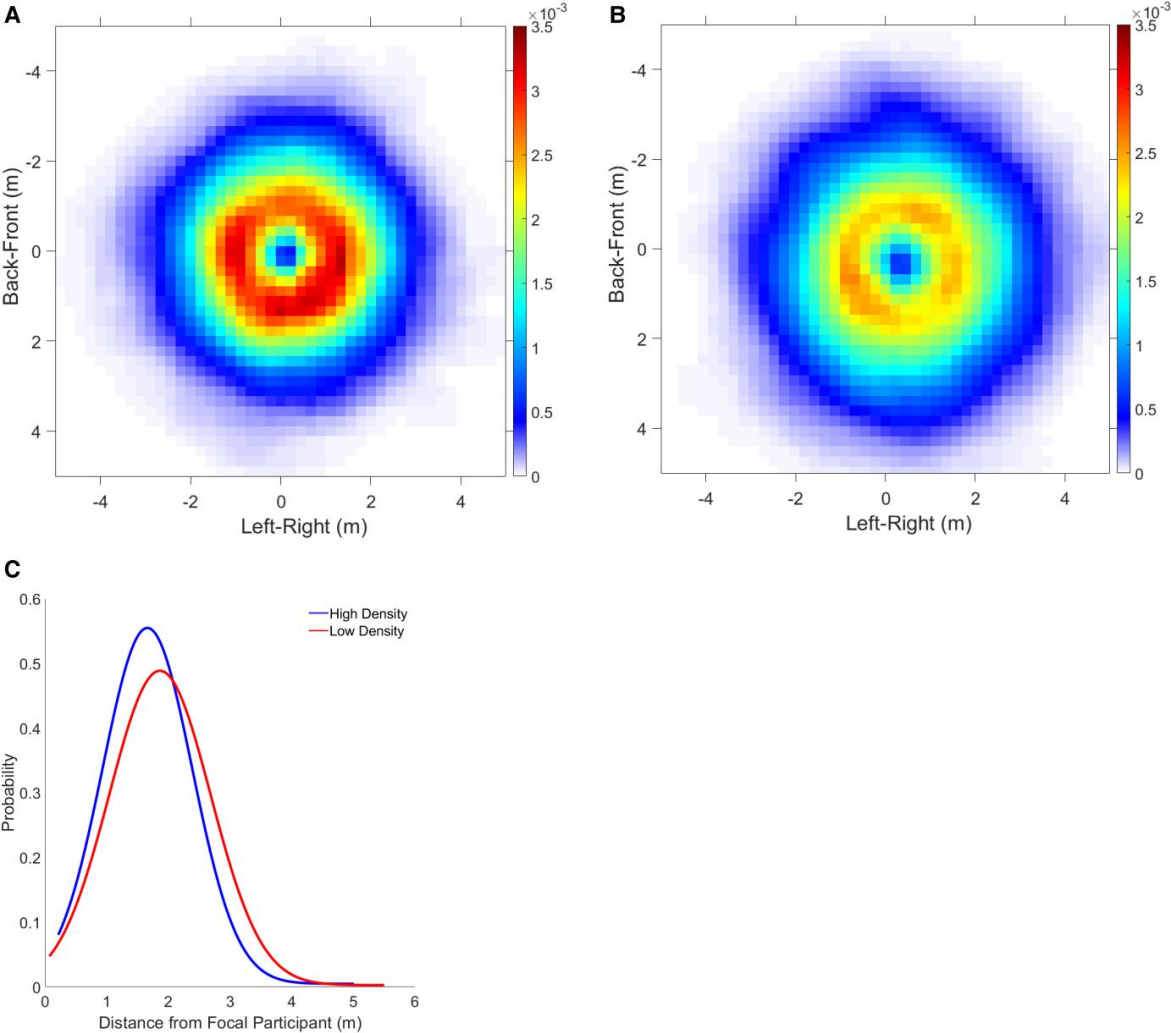
Occupancy probability density functions (PDFs) for all human swarm trials, plotted relative to the focal participant nearest the centroid of the swarm. A) High-density condition (mean 2.1 p/m^2^), six trials, 4.9 min of data. B) Low-density condition (mean 1.7 p/m^2^), six trials, 6.2 min of data. Color temperature in the heat maps represents the discrete probability density of observing a participant in each 0.2 m × 0.2 m cell, with focal participant *p* at the origin, heading upward. Larger area of hot reds in A) confirms the density manipulation. C) 1D PDF of occupancy as a function of distance from the focal participant in the high- (blue) and low- (red) density conditions.

We first checked that the density manipulation was successful by plotting a discrete probability density function for occupancy in each condition (Fig. 5). A shift in color temperature between panels is apparent, indicating that a greater density was maintained in the high condition (Fig. 5A, hot reds) than the low condition (Fig. 5B, cooler oranges and yellows). The mean measured density (participants per square meter in every frame of data) was 2.10 ± 0.004 p/m^2^ (SEM) in the high condition and 1.72 ± 0.005 p/m^2^ (SEM) in the low condition. An LME regression analysis confirmed a significant effect of the high/low manipulation on measured density (*χ*^2^(1) = 5056.8, *P* < 0.001), although a significant interaction (high/low × time) indicated that the difference decreased over the course of a 2-min trial (*χ*^2^(1) = 2378.5, *P* < 0.001) (see Fig. S3).

**Fig. 6.**
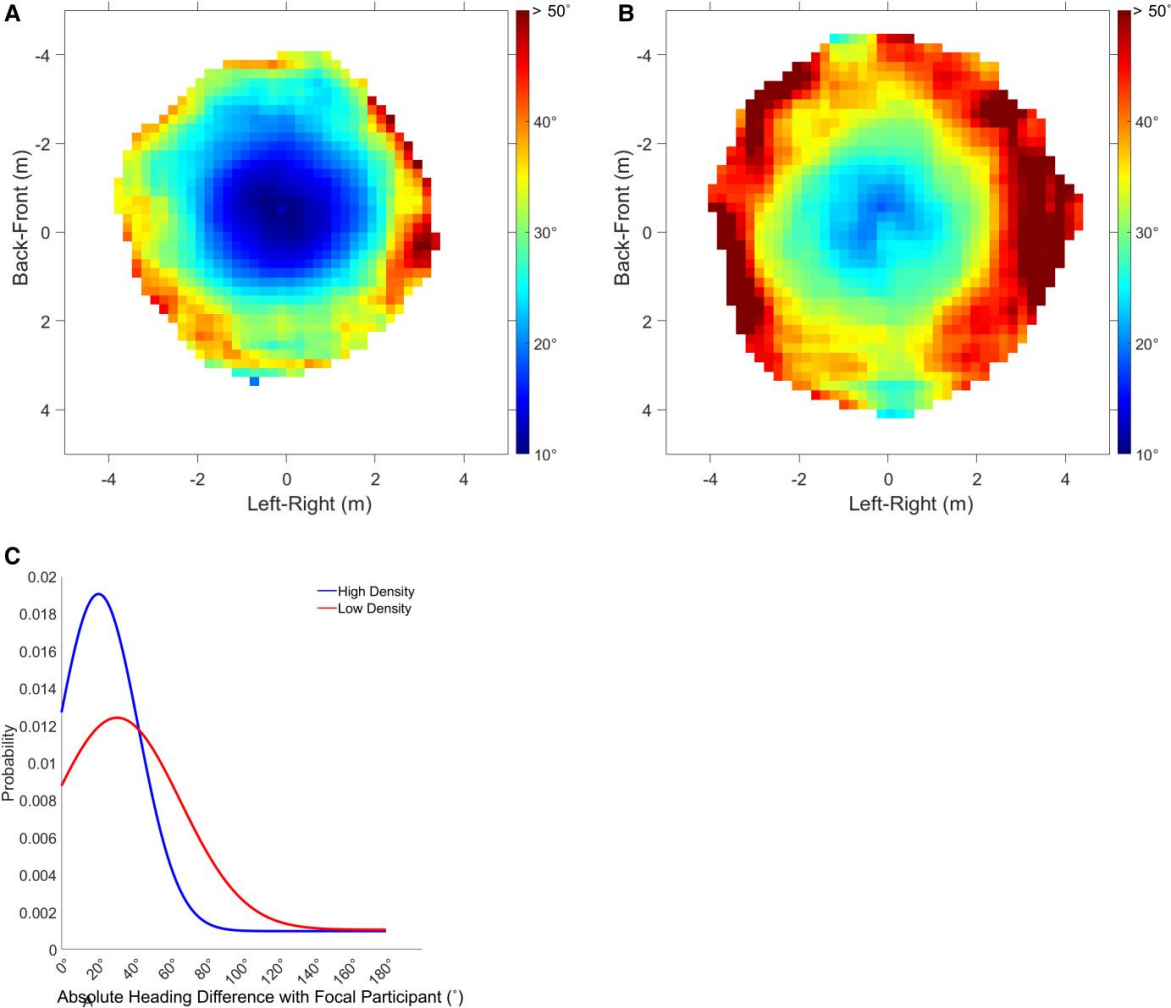
Heat maps of mean heading difference between the focal participant and each neighbor in human swarms. A) High-density condition, six trials, 4.9 min of data. B) Low-density condition, six trials, 6.2 min of data. Cells were only included in the heat map if they had at least 500 samples or 8.33 s worth of data. Color temperature represents the mean absolute heading difference $\left|\Delta \phi_{i,p}\right|$ between the focal participant *p* nearest the swarm's centroid (plotted at the origin, heading upward) and each neighbor *i* in the corresponding 0.2×0.2 m cell over all frames. Larger area of cold blues in A) indicates greater alignment in the high-density condition. C) 1D PDF of observations as a function of absolute heading difference with the focal participant, indicating greater alignment in the high-density (blue) than the low-density (red) condition.

To visualize the degree of alignment, we plotted heat maps of the mean absolute heading difference ($\left|\Delta \phi_{i,p}\right|$) between the “focal” participant *p* closest to the group centroid and each neighbor *i* (Fig. 6). The metric hypothesis predicts greater alignment in the high-density condition (25, 44), and indeed there is a larger region of cold blues (small heading differences) in the high-density (Fig. 6A) than the low-density (Fig. 6B) condition. On the topological hypothesis, one would expect the opposite, for the *n* nearest neighbors interact over longer distances in the low-density condition.

We then analyzed the dependence of alignment on metric distance. We computed the absolute heading difference between all pairs of participants *i* and *j* ($\left|\Delta \phi_{i,j}\right|$), pruned extreme cases unlikely to interact (heading difference >50° or distance >4.5 m; 21% of data), and then calculated the mean difference in consecutive 10-s time intervals and 0.25-m distance bins. An LME regression on heading difference in all trials confirmed a significant effect of metric distance (*χ*^2^(1) = 1482.1, *P* < 0.001); specifically, for every meter change in distance, there is a 5.45° ± 0.134° (SE) increase in the mean heading difference.

**Fig. 7.**
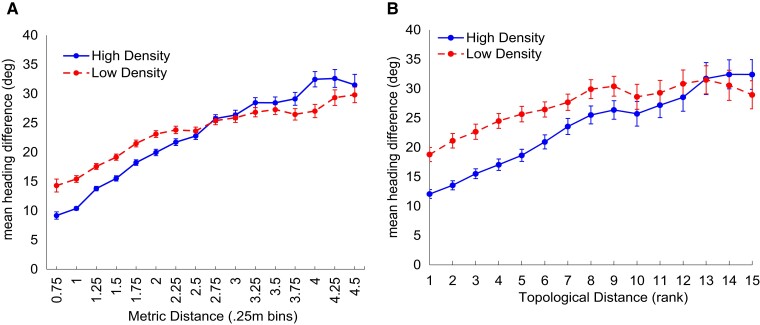
Mean absolute heading difference between all pairs of participants $\left|\Delta \phi_{i,j}\right|$ in human swarms, plotted as a function of A) metric distance and B) topological distance between *i* and *j*. The mean heading difference is smaller in the high-density condition (blue) than in the low density (red) in both plots (see text). Error bars represent the SEM, computed on the mean heading difference for all pairs of participants during each 10-s interval in all trials (six at each density). In A), the high- and low-density curves represent all heading differences less than 50° for pairs less than 4.5 m apart, yielding 4,093 estimates in the high- and 4,022 in the low-density condition. In B), the data were resorted to obtain heading differences less than 50° for each of the 15 nearest neighbors in each 10-s interval; metric distances ranged up to 8.3 m. This yielded 549 estimates in the high- and 528 in the low-density condition.

To test the neighborhood predictions, we sorted the heading differences ($\left|\Delta \phi_{i,j}\right|$) by metric distance (0.25 m bins) or by topological distance (ordinal rank) in each density condition. When plotted as a function of metric distance (Fig. 7A), the mean heading difference is smaller (greater alignment) in the high-density condition (blue curve) than the low-density condition (red curve). An LME regression on heading difference confirmed the density effect (*χ*^2^(1) = 6.51, *P* = 0.011), with a mean difference of 5.73° ± 1.37° (SE) between the high and low conditions. This finding is consistent with the metric hypothesis but contrary to the topological hypothesis. However, the interaction between density and distance was also significant (*χ*^2^(1) = 83.56, *P* < 0.001), and the two curves cross at a distance of 2.75 m, when the mean heading difference reaches 25°. At farther distances, the heading difference becomes larger at high density than low density, inconsistent with the metric hypothesis. This unexpected pattern is consistent with a visual neighborhood, for as distance increases, there are more completely occluded neighbors in the high- than the low-density condition (29) (see Fig. S4). Because a pedestrian is not influenced by occluded neighbors, the mean heading difference becomes larger in the high-density condition.

When replotted as a function of topological distance in Fig. 7B (after repruning cases with heading difference >50° or rank >15; 17% of data), the mean heading difference is again smaller in the high-density (blue curve) than the low-density (red curve) condition. This result indicates greater alignment between two neighbors who are physically closer but ordinally equidistant, contradicting the topological hypothesis. An LME regression revealed that the density effect is significant (*χ*^2^(1) = 9.26, *P* = 0.002) as is the interaction (density × ordinal rank) (*χ*^2^(1) = 19.39, *P* < 0.001). (See Table S3.)

##### Conclusion

The swarm experiment shows that heading alignment in real human crowds depends on density, whether plotted as a function of metric or topological distance. This finding provides decisive evidence against a topological neighborhood. The main effect of density is consistent with a metric neighborhood, but the density × distance interaction supports a visual neighborhood.

## Discussion

Previous reports of collective motion in animal groups have found that some species, like starlings, are governed by topological interactions that depend on ordinal distance, while others, like chimney swifts, obey metric interactions that depend on physical distance. The present research provides the first evidence that the neighborhood of interaction in human crowds is not topological, depends on metric distance, and is best explained by visual information. The metric hypothesis predicts that varying density will affect the strength of interaction, because neighbor influence is distance dependent. In contrast, the topological hypothesis predicts that varying density will have no effect, because neighbor influence only depends on ordinal rank. The visual hypothesis predicts that responses will be influenced by both density, which reflects metric distance, and visibility, which reflects ordinal rank.

In three experiments, we found that alignment reliably depends on density, specifically the proximity of perturbed and unperturbed neighbors. When random neighbors were perturbed, there was a stronger heading response at high density as the number of perturbed neighbors increased (also a stronger speed response). Conversely, when only the nearest neighbors were perturbed, there was a stronger heading response at *low* density, for unperturbed neighbors were farther away and exerted less influence. Measurements of human swarms also revealed a significant effect of density: we observed greater alignment at high density, regardless of whether the data were plotted as a function of metric or topological distance. The pattern of data thus qualitatively rules out a topological neighborhood, is in the expected direction for a metric neighborhood, but is more closely predicted by a visual neighborhood.

The visual neighborhood is determined by two factors, derived from the viewpoint of a pedestrian embedded in a crowd (23). First, when a neighbor changes heading direction or speed, this generates corresponding optical motions in the pedestrian's field of view. These optical velocities decrease with metric distance in accordance with Euclid's law. Second, near neighbors tend to partially occlude far neighbors, such that visibility decreases with both ordinal rank and metric separation in depth. The neighborhood's range of interaction corresponds to the distance at which nearer neighbors completely occlude all farther neighbors and thus varies dynamically with changes in density and visibility.

The visual model not only explains the density effects observed in the present experiments, it predicts the data in the second experiment much better than the metric model (Fig. 4). Whereas the omniscient metric model describes the decay with distance using a fixed exponential function, the visual model explains this distance dependence based on Euclid's law and the geometry of occlusion. Because it is sensitive to variation in neighbor distance and visibility, the model generalizes to crowds with different densities and distributions of neighbors. The present results thus provide critical support for a visual neighborhood in human crowds.

These findings have important implications for modeling crowd behavior and evacuation dynamics. Natural variation in crowd density ranges from 0 to 4 p/m^2^ before physical contact (45, 46), an order of magnitude more than the present manipulations, implying that topological models would generate large errors in crowd simulation. Metric models neglect the effects of density on visual occlusion and would also produce significant errors (23). Pursuing a visual model promises more realistic simulations of crowd dynamics.

It is possible that previously observed topological and metric interactions also have a visual basis. Notably, flocks of starlings and chimney swifts appear to have a different structure. Starlings (18) maintain a spatial configuration by keeping a near neighbor in four visual directions in the field of view (±90° azimuth and ± 45° elevation). The nearest neighbor in each quadrant would project the largest image and tend to occlude farther neighbors in the same direction; with some positional drift, this would yield a topological neighborhood of four to eight neighbors, consistent with the data. In contrast, roosting chimney swifts (30) tend to align their velocities, and alignment decreases gradually with metric distance, from 1.4 to 4–5 m. Heading responses in swifts might thus be governed by the same optical variables as in humans (Eq. 3), which decrease with metric distance and occlusion. Thus, nominally “topological” and “metric” neighborhoods could be a consequence of a visual neighborhood of interaction.

We conclude that the neighborhood of interaction follows naturally from the laws of optics. The influence of visible neighbors decays with metric distance due to Euclid's law and is further reduced by visual occlusion due to ordinal rank until the range of interaction is limited by complete occlusion. Previously observed “metric” and “topological” interactions may thus be consequences of a visual neighborhood.

## Materials and methods

### Virtual crowd experiments

#### Participants

Ten participants (5M and 5F) completed the first experiment, and 12 participants (7M and 5F) completed the second experiment; one additional participant was removed from the latter due to tracker error during data collection. All participants had normal or corrected-to-normal vision and none reported having a motor impairment. The research protocol was approved by Brown University's Institutional Review Board, in accordance with the principles expressed in the Declaration of Helsinki. Informed consent was obtained from all participants, who were paid for their participation.

#### Equipment

The experiments were conducted in the Virtual Environment Navigation Lab (VENLab) at Brown University. Participants walked freely in a 12 m × 14 m tracking area, while viewing a virtual environment in a wireless stereoscopic head-mounted display (Oculus Rift DK1, Irvine CA; 90°H × 65°V field of view, 640×800 pixels per eye, and 60 Hz refresh rate). Head position and orientation were recorded with a hybrid inertial/ultrasonic tracking system (IS-900, Intersense, Billerica, MA) and used to update the display. The frame rate in the first experiment varied between 30 and 60 Hz, as did the tracker sampling rate; in the second experiment, the frame rate and sampling rate were constant at 60 Hz. The measured display latency varied between 50 and 67 ms.

#### Displays

The virtual environment consisted of a ground plane with a grayscale granite texture and a blue sky. A green start pole and a red orienting pole (radius 0.2 m and height 3 m) appeared 12.73 m apart (the start pole was reduced to 1.3 m in the second experiment). The crowd consisted of 12 virtual humans (WorldViz Complete Characters) presented within the typical horizontal field of view (90°). In the first experiment only, 18 additional virtual humans were placed outside the field of view on two concentric circles to enhance the sense of immersion if the participant turned their head. The human models were animated with a walking gait with randomly varied phase. The racially diverse virtual crowd contained equal numbers of men and women.

In the first experiment, the 12 manipulated neighbors were initially positioned on two 90° arcs with the participant at the center, symmetric about the participant's initial walking direction (toward the orienting pole). The arc radii were *r* = 1.5 and 3.5 m in the high-density condition or 3.5 and 7.5 min the low-density condition. Five neighbors were placed at equal intervals on the near arc and seven on the far arc. In the second experiment, the four nearest neighbors appeared at fixed initial distances (on arcs with *r* = 1.5, 1.7, 1.9, and 2.1 m), and the nearest two or all four of them were perturbed. The other eight neighbors appeared on separate arcs spaced 0.2 m apart in depth (*r* = 2.3, 2.5, … 3.7 m) in the high-density condition or 1 m apart in depth (*r* = 3.1, 4.1, … 11.1) in the low-density condition. The eccentricity *θ* of each neighbor was randomly selected from six equally spaced points on an 80° arc centered on the initial walking direction.

These initial positions were then jittered in polar coordinates, with the radial displacement Δ*r* randomly selected from a Gaussian distribution (*μ* = 0 m and *σ* = 0.15 m) and the angular displacement Δ*θ* from a separate Gaussian distribution (*μ* = 0° and *σ* = 8°). A different crowd configuration was generated for each trial; all participants received the same set of configurations, but virtual humans were randomly assigned to the positions.

During a trial, all virtual humans accelerated forward from a standstill (0 m/s) to a walking speed (1.0 m/s) over a period of 3 s following a sigmoidal function (cumulative normal, *μ* = 0 and *σ* = 0.5 s) fit to previous human data. They walked on parallel linear paths for 2 s, then the heading direction of a subset of the 12 neighbors was perturbed by ±10°, all to the right or to the left, over a period of 0.5 s, following a similar sigmoidal function (*μ* = 0 and *σ* = 0.083 s). They continued walking on new linear paths for another 8 s. In the second experiment, crowd speed was increased to 1.15 m/s, closer to participants’ preferred walking speed, and the display thus continued for 5.4 s.

The crucial manipulations were the following. In the first experiment, the perturbed subset (0, 3, 6, 9, or all 12 neighbors) was randomly selected from near and far neighbors. (The speed of the subset was similarly perturbed by ±0.3 m/s in a separate condition; see Supplementary Data S1.) In the second experiment, only the nearest neighbors (0, 2, or 4) were perturbed. The four nearest neighbors always appeared at the same distances, and the density manipulation only affected the distances of the eight other neighbors.

#### Procedure

Participants were instructed to walk as naturally as possible, to treat the virtual pedestrians as if they were real people, and to stay together with the crowd. On each trial, the participant walked to the green start pole and faced the red orienting pole. After 2 s, the poles disappeared and the virtual crowd appeared; 1 s later, a verbal command (“begin”) was played and the virtual crowd began walking. The display continued until the participant had walked about 12 m (a duration of 12 s in the first experiment and 10.4 s in the second); a verbal command (“end”) signaled the end of the trial. There were two practice trials to familiarize the participant with walking in the virtual environment. During this time, the participants could adjust the inter-ocular distance (IOD) of the head mounted display (HMD) so that the display was clearly visible.

#### Design

*First experiment*: 5 perturbed subsets (0, 3, 6, 9, and 12 neighbors) × 2 densities (high and low) × 2 perturbations (heading and speed). There were 8 trials per condition, for a total of 160 trials presented in a randomized order in two 1-h sessions. The 80 heading perturbation trials are reported in the text, and the results from the 80 speed perturbation trials appear in Fig. S1. *Second experiment*: 3 perturbed subsets (0, 2, and 4 nearest neighbors) × 2 densities (high and low). There were 16 heading perturbation trials per condition, yielding a total of 96 trials presented in a randomized order in a 1-h session.

#### Data processing

For each trial, the time series of head position in the horizontal (*X*–*Y*) plane were filtered using a forward and backward fourth-order low-pass Butterworth filter to reduce the effects of oscillations due to the step cycle and occasional tracker error. Time series of heading direction and walking speed were then computed from the filtered position data. A 0.6-Hz cutoff was used for computing heading to reduce lateral oscillations on each stride, while a 1.0-Hz cutoff was used for computing speed to reduce anterior–posterior oscillations on each step. Right and left turn trials were collapsed by multiplying the heading angle on left turns by −1. Speed-up and slow-down trials were collapsed by first (i) normalizing walking speed by subtracting the walking speed of unperturbed crowd (1 m/s) from participants’ speed time series and then (ii) multiplying the normalized speed on slow-down trials by −1, to yield the absolute change in speed. Final heading and final speed were then computed as the average value during the last 2 s of each trial (4 to 6 s postperturbation in the first experiment and 2.4 to 4.4 s in the second). To further mitigate the effect of gait oscillations, a mean time series was computed for each participant in each condition. Dependent measures included the mean final heading, and the mean time series of heading, for each participant in each condition (and the same for absolute speed change in the first experiment).

#### Statistical analysis

We took a LME regression approach, using the *fitlme* function (maximum likelihood approximation) in MATLAB (R2019b). The dependent variable (e.g. heading) is regressed on predictor variables that may include categorical fixed effects (e.g. density), continuous fixed effects (e.g. time), and random effects (e.g. subjects, with unique intercepts). The residuals were inspected for any obvious heteroscedasticity or deviations from normality. Main effects and interactions were tested by comparing models in a step-down procedure that removes tested terms from the full model, using likelihood ratio *χ*^2^ tests. Slopes are described for significant effects.

We performed two LME regression analyses: one on mean final heading and the second on the mean heading time series (see Fig. 3, Table S1 for Experiment 1, and Table S2 for Experiment 2). Parallel analyses were performed on the speed data (see Fig. S1 and Table S1C).

### Human swarm experiment

#### Participants

One group of 10 participants, one group of 16 participants, and one group of 20 participants were tested in separate sessions as part of a larger study. The protocol was approved and informed consent was obtained as before, and participants were paid for their time.

#### Equipment

Head position was recorded in a large hall with a 16-camera infrared motion capture system (Qualisys Oqus, Buffalo Grove, IL) at 60 Hz. The tracking area (14 m × 20 m) and starting boxes were marked on the floor with colored tape. Each participant wore a bicycle helmet with a unique constellation of five reflective markers on 30–40-cm stalks.

#### Procedure

Participants were instructed to walk about the tracking area at a normal speed, veering randomly left and right, while staying together as a group, for 2-min trials. Participants began each trial in shuffled positions in one of the starting boxes, corresponding to high- and low-density conditions: a 2 × 2 m or 3 × 3 m box for the 10-person group, a 3 × 3 m or 4 × 4 m box for the 16-person group, and a 4 × 4 or 7 × 7 m box for the 20-person group. At a verbal “go” signal, they began walking for 2 min, until a “stop” signal. Each group received two trials in each density condition.

#### Design

There were three groups (*N* = 10, 16, and 20) and 2 initial densities (high and low). There were two trials per condition, yielding a total of 12 trials with 24 min of raw data.

#### Data processing

The 3D position of the centroid of the markers on each helmet was reconstructed on each frame using a custom algorithm. Due to limits on the viewing volume and infrared reflections in the hall, there were many tracking errors, such that 100% of the helmets were recovered in 45% of all frames. The time series of head position in the 2D horizontal (*x*,*y*) plane was processed and filtered as before, and the heading direction of each helmet was computed on each time step in which it was successfully tracked; speed did not vary appreciably and was not analyzed further.

We measured the density of the swarm in each frame as the number of participants per square meter (p/m^2^). Because this measurement depends on knowing the position of every participant, only frames in which 100% of the helmets were recovered were used in this analysis. We first computed a 2D boundary around the (*x*,*y*) positions of the participants using MATLAB's *boundary* function, with the “shrink” parameter set to the default value of 0.5; it ranges from 0 to 1, where 0 produces the convex hull and 1 a concave boundary that hugs the points tightly. We then computed the area of that polygon using the *polyarea* function and finally calculated density by dividing the number of participants in that frame (*p*) by the area of the polygon (m^2^). This method is sufficient to capture the relative density in low and high conditions for each group (with constant *N*). For a robust estimate, we averaged the measured density of all frames in each 10-s segment on each trial. The mean density of the trials in each time bin in the high and low conditions is plotted as a function of time bin in Fig. S3; the error bars represent the SE of the trial means in each time bin, where each bin includes one to four samples.

### Simulation methods

Individual trials from the virtual crowd experiments were simulated in MATLAB using the Runge–Kutta method (*ode45* function). The participant's position, heading, and speed 2 s before the perturbation were taken as the initial conditions. For the metric model (Eqs. 1a and 1c) and topological model (Eqs. 1a and 2) simulations of heading, the input on each time step was the distance and heading of the virtual humans in the participant's field of view and the recorded time series of the participant's walking speed in that trial; the output was a time series of the agent's heading. When simulating speed (Eqs. 1b and 1c, first experiment only), the input was the distance and speed of the virtual neighbors, and the output was a time series of the agent's speed (Fig. S1). For the visual model (Eq. 3), the input was the angular velocity, optical expansion rate, eccentricity, and visibility of each virtual human, which were calculated from their positions on each time step. The outputs were time series of the agent's (*x*,*y*) position, heading, and speed.

#### Model comparisons

To compare the simulations with the human data, we first calculated the mean time series of heading (or speed) for each participant in each condition and for the corresponding model agent. We then computed the mean absolute error (MAE) between each model agent and the participant time series in each condition. Finally, we compared the models to one another by calculating Bayes factors (BF_vm_) based on the MAE between the model and each subject. Note that the variability in the final heading is very small for the models because gait oscillations and tracker error were not simulated, so we compare the model means with 95% CI for the human data in the figures.

#### Model performance benchmarks

The performance of any model is limited by the inherent noise in the human data due to gait oscillations and tracker error. To benchmark the lower bound on error, we estimated the fluctuations in heading when walking on a straight path by computing the RMSE between each participant's mean time series of heading on *control* trials (0 neighbors perturbed) and a heading of 0°. Conversely, to benchmark the upper bound on error—the failure of a model to respond to a perturbation—we estimated the error for a model that does not respond to the input by computing the RMSE between each participant's mean heading time series on *perturbation* trials and a heading of 0°. These benchmarks indicate the range of model performance, from the best possible performance given the noise in the data to the performance of a model that does nothing. Of course, the performance of a model that responds inappropriately would be even worse.

## Supplementary Material

pgad118_Supplementary_Data

## Data Availability

The data that support the findings of this study are openly available in the Brown Digital Repository at https://doi.org/10.26300/xk9b-5d55 (47).
